# Application of natural compounds in the treatment and prevention of prediabetes

**DOI:** 10.3389/fnut.2023.1301129

**Published:** 2023-11-30

**Authors:** Jie Chen, Li Jin, Mengyao Chen, Kai Xu, Qi Huang, Beihui He

**Affiliations:** ^1^The First Affiliated Hospital of Zhejiang Chinese Medical University (Zhejiang Provincial Hospital of Chinese Medicine), Hangzhou, China; ^2^School of Pharmaceutical Sciences, Zhejiang Chinese Medical University, Hangzhou, China

**Keywords:** prediabetes, natural compounds, oxidative stress, insulin resistance, gut microbiota

## Abstract

Prediabetes is an intermediate stage in the development of type 2 diabetes mellitus characterized by impaired fasting glucose and/or impaired glucose tolerance. Prediabetes generally has no obvious clinical symptoms, and most patients are found in health examinations or due to other diseases. Reactive hypoglycemia may indicate the possibility of early diabetes. Without effective preventive measures, prediabetes can progress to diabetes leading to serious public health problems. Therefore, early diagnosis and intervention are important. Many animal experiments and clinical trials have proven that natural compounds substantially improve glucose metabolism disorder. The active ingredients are mainly alkaloids, polysaccharides, saponins, terpenoids, flavonoids and polyphenols. Their mechanism of action mainly involves improved insulin sensitivity and insulin resistance, inhibited activity of alpha-glucosidase, antioxidant activity, anti-inflammatory, regulation of gut microbiota and activating of peroxisome proliferator-activated receptor-γ. This paper reviews the mechanisms of action of natural compounds on prediabetes and the status of related research.

## 1 Introduction

Diabetes is a chronic disease that seriously affects the quality of life of patients. It includes different types, of which type 2 diabetes (T2D) accounts for the majority. Prediabetes is an intermediate stage of glucose dysregulation (1) that consists of impaired fasting glucose (IFG) (6.1 mmol/L ≤ fasting plasma glucose<7.0 mmol/L), impaired glucose tolerance (IGT) (fasting plasma glucose<6.1 mmol/L and 7.8 ≤ 2-h fasting plasma glucose<11.1 mmol/L), or both. Both IFG and IGT have both insulin resistance and beta-cell defect, postprandial reactive hypoglycemia is also known as prediabetic reactive hypoglycemia because it may be associated with IFG and/or IGT (2). An estimated 280 million people currently have prediabetes. This number is projected to reach 548 million by 2045, accounting for 8.4% of global adult population (3, 4). Approximately 5 to 10% of patients with prediabetes will progress to diabetes without effective preventive measures each year (5). The prevention of diabetes is an urgent issue.

The pathogenesis of prediabetes is not fully understood and may involve insulin resistance (IR), chronic inflammatory responses, pancreatic beta cell dysfunction, oxidative stress, gut microbiota (6).

Lifestyle intervention is the first-line approach to prevent or delay T2D in adults with prediabetes (7). Drug therapy is necessary if lifestyle interventions fail (8). Although drug interventions such as metformin, α-glycosidase enzyme inhibitor, GLP-1 receptor agonist, SGLT2 inhibitor, thiazolidinedione can reduce the risk of diabetes in prediabetes (9, 10), but adverse reactions, such as diarrhea and abdominal distension, huge cost should become prehensive considered for long-term use (11). Natural compounds increasing attention owing to their safety, few side effects, and definite curative effects mainly involves improved insulin sensitivity and insulin resistance, inhibited activity of alpha-glucosidase, antioxidant activity, anti-inflammatory, regulation of gut microbiota (12). Natural compounds have good anti diabetes and prediabetes properties, mainly including phenolic compounds, flavonoids, terpenoids, alkaloids, glycosides, quinones, lactones and saponins (13).

This paper reviews the mechanisms of action of natural compounds on prediabetes and the related research status. The intent is to provide an important basis and new research mentality for the development and application of natural compounds.

## 2 Active ingredients of natural compounds

Natural compounds with varying biological and pharmacological activities for prediabetes include flavonoids, polyphenols, terpenoids, alkaloids, polysaccharides, quinones, lactones, and saponins. Progresses of natural compounds in anti prediabetes *in vivo*, *in vitro* and clinical trials have shown in Table 1.

**Table 1:** Progress of natural compounds in anti prediabetes *in vivo*, *in vitro* and clinical trials.

Classification	Drug treatment	Subjects	Sample size	Dosage time of intervention	Results
Alkaloids	Sangzhi alkaloids	Prediabetic mice	20	50 mg·kg^−1^, 6 weeks	FBG, FINS, TC decrease (14)
	Berberine	IGT rats	25	100 mg/kg/d, 3 weeks	Diabetes developed in all rats in the IGT group, but only 30% of rats in the berberine group (15)
Polysaccharides	Astragalus polysaccharide	Insulin cells treated with high glucose and palmitic acid	Cells	50, 100, and 200 μg/mL	Proliferation and insulin secretion improve (16)
	Guar gum	Prediabetes, moderately well-controlled T2DM patients	24	5 g, 5 days	3-h glucose measurement showed a significant reduction at all time points except 120 min recording the highest decrease at 45 min (17)
Saponins	Saponins of *panax ginseng*	Diabetic rat model was induced in SD rats by streptozocin administration	21	40, 80 mg/kg, 26 days	SD rats pretreated with saponins of *panax ginseng* did not develop into diabetes (18)
	Saponin of litchi seed	Hyperlipidemia-fatty, liver-insulin resistance rats	15	0.05, 0.1 g·kg^−1^, 6 weeks	FBG, 2hPG, TC, TG, LDL-C decrease (19)
	Gymnemic acid	IGT patients	30	300 mg bid,12 weeks	HbA1c, 2 h PG decrease (20)
Terpenoids	Oleanolic acid	Prediabetes SD rats	36	80 mg/kg,32 weeks	Lipid metabolism improved, plasma proinflammatory cytokines decrease (21)
	Ursolic acid	Insulin resistant rats induced by high fat diets	60	150, 300 mg/kg/d, 17 weeks	Fasting serum insulin, HOMA – IR, serum total cholesterol, triglyceridereduce (22)
	Bredemolic acid	Skeletal muscle cells	Cells	12.5 mmoL/L	TOAC, glucose utilization improve (23)
	Oleanolic acid	Prediabetes individuals	176	30 mg/day	Lipid metabolism improved, plasma proinflammatory cytokines decrease (24)
	Geniposide	Prediabetic patients	86	TCM, 3 months	HbA1c, FBG, 2 h PG decrease (25)
	Steviol glycoside	Prediabetic women	24	125 mg sweetener,14 days	FPG decrease (26)

### 2.1 Alkaloids

In one study, Sangzhi alkaloid was administered for 6 weeks to prediabetic HFC mice. The intervention significantly lowered fasting blood glucose (FBG), random blood glucose, fasting insulin, and total cholesterol (TC); improved glucose and lipid metabolism and IR; and ameliorated glucose tolerance through increasing the insulin secretion multiple by 51.3% after 15 min of glucose stimulation (*p* = 0.07) (14). Wang et al. (15) established an IGT rat model using a high-energy diet. After 3 weeks of berberine treatment, diabetes developed in all rats in the model group, but in only 30% of rats in the treatment group. The FBG level in the berberine group (5.03 ± 1.26 mmol/L) was significantly lower than that in the IGT group (*p* < 0.05). These results showed that berberine delayed the progression of prediabetes to T2DM in IGT rats by improving the structure of the gut microbiota and intestinal permeability.

### 2.2 Polysaccharides

Deng et al. (16) used astragalus polysaccharide as an intervention to treat insulin cells exposed to high glucose and palmitic acid. Astragalus treatment ameliorated proliferation and insulin secretion in dysfunctional cells partially by upgrading the expression of microRNA (miR)-149-5p and miR-136-5p. Guargum is a natural polysaccharide extracted from the seeds of the *Cyamopsistetragonolobus* guar plant. Clifton et al. (17) recruited prediabetic and moderately well-controlled T2D patients who consumed a drink consisting mainly of guar gum for 2 days or cold water on the other 2 days before breakfast. Three-hour glucose finger prick test measurements demonstrated an obvious decrease at every time point, peak finger prick glucose was reduced by 2.1 mmol/L at 45 min (*p* < 0.0001). Average finger prick glucose over 3 h was reduced by 0.8 mmol/L (*p* = 0.0003).

### 2.3 Saponins

The preventive administration of saponins of *panax ginseng* was given to Sprague Dawley (SD) rats, then diabetic rat model was induced in SD rats by streptozocin administration. The outcome showed that SD rats pretreated with saponins of *panax ginseng* did not develop diabetes (18). Guo et al. (19) administer edlitchiseed saponin to hyperlipidemia-fatty, liver-insulin-resistant rats. After administration of litchi seed saponin, the FBG and 2 h PG significantly decreased (*p* < 0.05), IGT improved, and the levels of TC, triglyceride (TG), and low-density lipoprotein-cholesterol (LDL-C) decreased, with improved insulin sensitivity. Martínez et al. (20) studied the administration of *Gymnema sylvestre* leaf extract containing at least 23 saponins to an IGT population. The extract significantly reduced glycated hemoglobin (HbA1c) levels (5.8 ± 0.3% vs. 5.4 ± 0.4%, *p* = 0.025), and 2-h PG levels (9.1 ± 1.2 vs. 7.8 ± 1.7 mmol/L, *p* = 0.003) after the oral glucose tolerance test compared to baseline, thereby improving insulin sensitivity.

### 2.4 Terpenoids

Gamede et al. (21) established prediabetes in SD rats by feeding them a high-carbohydrate high-fat diet. The prediabetic rats were then treated with oleanolic acid for 12 weeks. Oleanolic acid upgraded lipid metabolism by recovering LDLs and high-density lipoproteins, while decreasing immune cell and platelet counts. Oleanolic acid also reduced plasma level of proinflammatory cytokines, including containing tumor necrosis factor-1β and tumor necrosis factor-α. Immune activation markers, such as fibrinogen, C-reactive protein, and CD40L, were also reduced after the administration of oleanolic acid. According to a clinical trials related to Oleanolic acid (OA), 176 prediabetes individuals were recruited, 55 mL/day of olive oil rich in OA (equivalent to a dose of 30 mg OA/day) was given, the results showed that the intake of olive oil rich in OA can reduce the risk of diabetes in pre diabetes patients, thus promoting the use of OA in new functional foods and drugs to prevent individuals at risk of diabetes (24). Wei (25) recruited 86 prediabetic patients and randomly assigned them to control and treatment groups. Both groups underwent 3 months of diet control and exercise. Those in the treatment group additionally received Jiawei Yueju Decoction, whose main active ingredients include geniposide, the main components of iridoids, which had been demonstrated to reduce blood glucose levels in mice in a dose-dependent manner. After 3 months of treatment, FBG, 2-h PG and HbA1c decreased significantly in the treatment group (*p* < 0.05), with reductions in in TG, TC, and LDL-C levels that were more obvious than the reductions in the control group (*p* < 0.05). Mayasari et al. (26) observed the effect of a mixed herbal drink consisting of *Stevia rebaudiana* (steviol diterpene glycosides are natural compounds of *S. rebaudiana* leaves that impart sweetness) and *Hibiscus sabdariffa* (rosella tea) on glucose metabolism in women with prediabetes. Half the women consumed the mixed herbal drink and the other half consumed a control drink lacking steviol diterpene glycosides. After 14 days, a significant decrease in FPG levels was observed in the treatment group (from 111.25 ± 7.20 mg/dL to 88.58 ± 13.19 mg/dL; *p* < 0.01). Zhang et al. (22) established insulin resistant rats induced by high fat diets, after 4 weeks and 8 weeks administration, they found ursolic acid could markedly reduce the levels of fasting serum insulin, HOMA-IR, serum total cholesterol, triglyceride, and improve the insulin sensitive index, which means ursolic acid could improve insulin resistance which is the main reason for prediabetes. In previous studies, bredemolic acid has been reported as an antidiabetic agent that improves glucose uptake, ameliorates insulin resistance, and oxidative stress in the liver, heart, kidney, and skeletal muscle of prediabetic rats. Insulin resistance was induced in the skeletal muscle cells after 4 h of exposure to palmitic acid (0.5 mmol/L), and then treated with either insulin (4 μg/mL) or bredemolic acid (12.5 mmol/L) or with both. The results showed that bredemolic acid significantly improved TOAC and promoted glucose utilization via attenuation of lipid peroxidation and increased glycogen formation in the insulin-resistant cells, respectively (23).

### 2.5 Flavonoids

Cesar et al. (27) treated prediabetic patients with the nutraceutic alerythromycin, a compound of citrus flavonoids (naringin, hesperidin, eriocitrin, and didymin). Glycemia was reduced and glucagon-like peptide-1 (GLP-1), butyrate and acetate production increased. 16rRNA gene sequencing showed that eriomin provoked the growth of *Subdoligranulum* and *Bacteroides*, which are related to superior host glycemic metabolism. Xian et al. (28) collected data from middle-aged and elderly women with prediabetes and found that tea made from guava leaves, which contain flavonoids as one of the main active ingredients, was one of the most effective hypoglycemic treatments along with diabetes education in the 3-month treatment period. Levels of FBG, 2 h-PG, and HbA1c in these patients were significantly better than the levels in the control group (*p* < 0.05). Finally, the positive effect of quercetin on glucose homeostasis by reducing FPG levels and Homeostatic Model Assessment for IR (HOMA-IR) values has been reported (*p* < 0.001) (29). Quercetin is a major member of the flavonoid family found in various vegetables and fruits.

### 2.6 Polyphenols

Chen et al. (30) randomly patients with newly prediabetes or T2D into control and treatment groups, using the patient’s own body as a control. The treatment group was treated with Elitea Essence, a Pu-erh tea extract whose main ingredient is polyphenols. Both groups underwent a 100-g steamed-bread meal test. C-peptide, insulin, and GLP-1 levels were significantly higher following treatment (*p* < 0.05), indicating that Elitea Essence could enhance postprandial insulin secretion from pancreatic β cells, and suppress glucagon secretion, thereby lowering postprandial blood glucose levels of IGT. Chuengsamarn et al. (31) recruited 240 patients with prediabetes. They received placebo capsules or curcumin. After 9 months of treatment, none of the subjects in the curcumin-treated group were diagnosed with T2DM, while a small number of subjects in the placebo control group were diagnosed with T2DM. In addition, the curcumin group showed a superior overall function of β-cells, with lower C-peptide (1.7 vs. 2.17, *p* < 0.05) and higher HOMA-β (61.58 vs. 48.72, *p* < 0.01). The curcumin-treated patients displayed higher adiponectin levels and lower HOMA-IR values compared with the control group (3.22 vs. 4.04, *p* < 0.001). Lignans are natural compounds isolated from nutmeg. Finite evidence indicates the short- and long-term efficacy of lignans in managing at least three of seven conditions (central obesity, hyperglycemia, hypertension, dyslipidemia, pro-thrombosis, oxidation, and inflammation) prior to and following the onset of T2D. Large multicenter clinical trials are required to determine the potential of lignans in prediabetes and T2D (32).

## 3 Mechanism of action of natural compounds

### 3.1 Improve insulin sensitivity and IR

Guava leaf tea, which contains flavonoids as the main active ingredient, is one of the most effective hypoglycemic therapies. Consumption of this tea can increase glucose uptake and utilization by the peripheral tissues, improve insulin sensitivity, protect islet β cells, and ultimately meliorate the metabolism of glucose (28). In one study, treatment with guava leaf tea improved blood sugar levels in middle-aged and elderly women with prediabetes (28). Curcumin, the main active part of the spice turmeric kurkum, is collected from the rhizome of the tropical plant *Curcuma longa* L. Curcumin is recommended to prevent the progression of prediabetes to diabetes based on its proven safety and efficacy. The efficacy mainly involves improving β-cell function, preventing β-cell apoptosis, and reducing IR (31). Chlorogenic acid is a major polyphenol found in green coffee beans. It seems to have hypoglycemic functions resembling those of metformin by improving glucose uptake and IR without side effects, thus decreasing FPG and increasing insulin sensitivity in patients with IGT (33). Fenugreek powder is useful in reducing the cumulative incidence rate of prediabetes. After treatment with fenugreek powder in a prediabetic population, the development of diabetes was definitely related to serum insulin and negatively associated with HOMA-IR. The main mechanism may involved decreased IR (34). *Ilex paraguariensis* is a perennial tree that grows in southern Brazil. Extracts have shown potential benefits to human health, such as hypoglycemic effects, weight reduction, and high antioxidant activity, due to the presence of various bioactive compounds, including polyphenols, saponins, and flavonoids. Treatment reportedly reduced FPG and HOMA-IR levels by improving IR in patients with prediabetes (35). Nuciferine is a natural compound found in lotus leaves. The variety of physiological activities and pharmacological effects include antidiabetic and antioxidant effects. Following consumption, nuciferine becomes densely distributed on the intestinal wall, creating a layer that can retain fat. This activity restricts the absorption of fat, inhibits fat accumulation, reduces body weight, and improves insulin sensitivity, thus preventing or delaying the progression from prediabetes to diabetes (36). *Aspergillus dracunculus* is a representative medicinal plant. Estragole is the main compound responsible for the antihyperglycemic properties of *A. dracunculus*. The toxicity of estragole may be removed by the preparation of an ethanol extract. One study found that the ethanol extract of *A. dracunculus* in patients with IGT decreased the area under the receive operating characteristic curve of insulin and total insulin secretion (37). Remarkable reductions in HbA1c and blood pressure were evident, due to increases of insulin sensitivity and insulin signaling. These increases resulted from the activated activity of the insulin receptor substrate associated with AKT1, phosphoinositide 3-kinase, and phosphorylation of AKT in skeletal muscle.

### 3.2 Inhibition of alpha-glucosidase activity

Sangzhi alkaloids are highly selective disaccharidase inhibitors that prevent the enzymatic hydrolysis of carbohydrates by inhibiting α-glucosidase activity, which controls postprandial elevation in blood glucose. Long-term treatment may be beneficial to delay the progression prediabetes to diabetes (14). In one study, the long-term administration of this drug effectively improved glucose and lipid metabolism in prediabetic mice. Timosaponin AIII is a steroidal saponin extracted from *Anemarrhena asphodeloides.* It inhibits α-glucosidase activity, which can postpone or decrease the digestion and absorption of carbohydrates, reduce the postprandial blood glucose level, reduce blood sugar fluctuations, and delay or prevent diabetes (38). Anthocyanins are glycosides of anthocyanidins. Anthocyanins are a subclass of flavonoids that are responsible for the deep blue, purple, and red colors of many vegetables and fruits. Anthocyanidins and their glycosides can inhibit α-amylase and α-glucosidase activities and improve the expression of glucose transporter 4 in cell membranes. These activities improve the lipid profile and promote glucose uptake, which can delay the onset of diabetes (39).

### 3.3 Decreased oxidative stress

Increased oxidative stress and decreased antioxidant activity are associated with prediabetes and diabetes. Anthocyanins have shown promising antioxidant and antidiabetic effects (39). Flavonoids in buckwheat decrease high glucose-induced oxidative stress. Flavonoids also significantly inhibit the production of reactive oxygen species (ROS) and improve glucose consumption and glycogen content in HepG2 cells, thus showing appreciable antioxidant and antidiabetic characteristics (40). Ginseng contains various compounds. Among these, ginsenosides have been implicated in its hypoglycemic action. The preventive effect of saponins from *Panax ginseng* is related to the antioxidative stress activity involving decreased malondialdehyde (MDA) and ROS levels, and increased superoxide dismutase (SOD) levels, thus preventing diabetes onset and retarding its progression (41).

### 3.4 Anti-inflammatory effect

Chinese yam polysaccharides reduce the body weight of obese mice. The polysaccharides also ameliorate IR by reducing serum inflammatory factors interleukin (IL)-10, IL-1β, and leptin, and increasing insulin sensitivity (41). OA may offer an alternative to hinder the progression of prediabetes to diabetes. A decrease in proinflammatory cytokine levels indicates that OA has anti-inflammatory properties involving a weakened innate immune cell response and inhibited production of proinflammatory cells in prediabetic individuals (21).

### 3.5 Regulation of gut microbiota

Eriomin is a mixture of the citrus flavonoids hesperidin, naringin, eriocitrin and didymin. Eriomin treatment of prediabetic patients resulted in significantly increased beta diversity, indicating an influence on the gut microbiota (27). Acetate and butyrate production were increased and the growth of *Bacteroides* and *Suboligranulum* was stimulated. *Bacteroides* comprise the largest portion of the intestinal microbiota and are latent colonizers of the colon, with some species generating acetic and propionic fatty acids. *Suboligranulum* species are associated with improved glycemic metabolism in the host. Finally, the demonstration that eriomin improves the composition and metabolism of the gut microbiota indicates its potential value as a treatment of prediabetes (27). Berries that are rich in polyphenol regulate intestinal microflora ecology by reversing/reducing the high-carbohydrate/high-fat-induced alterations in glucose metabolism-related pathways. These events result in a decline in the *Bacteroidetes/Firmicutes* ratio, short-chain fatty acids, and organic acid-producing microflora (42). Berberine is a natural product belonging to a group of alkaloids presents in many medicinal plants. In one study, berberine appeared to postpone the progression of prediabetes to T2D in ZDF rats by recovering the structure of the gut microbiota and intestinal permeability, as well as the abundance of gram-negative bacteria (15). *Dendrobium officinal*e polysaccharides are the major active ingredients in *D. officinale*. The polysaccharides serve as prebiotics and have various biological activities, including hypoglycemic activity. Finally, treatment with *D. officinale* polysaccharides decreased the *Bacteroidetes/Firmicutes* ratio and increased the abundance of *Bifidobacterium* and *Lactobacillus*, thereby preventing the progression of prediabetes to diabetes (43).

### 3.6 Peroxisome proliferator-activated receptor-γ (PPAR-γ) activation

**Figure 1:**
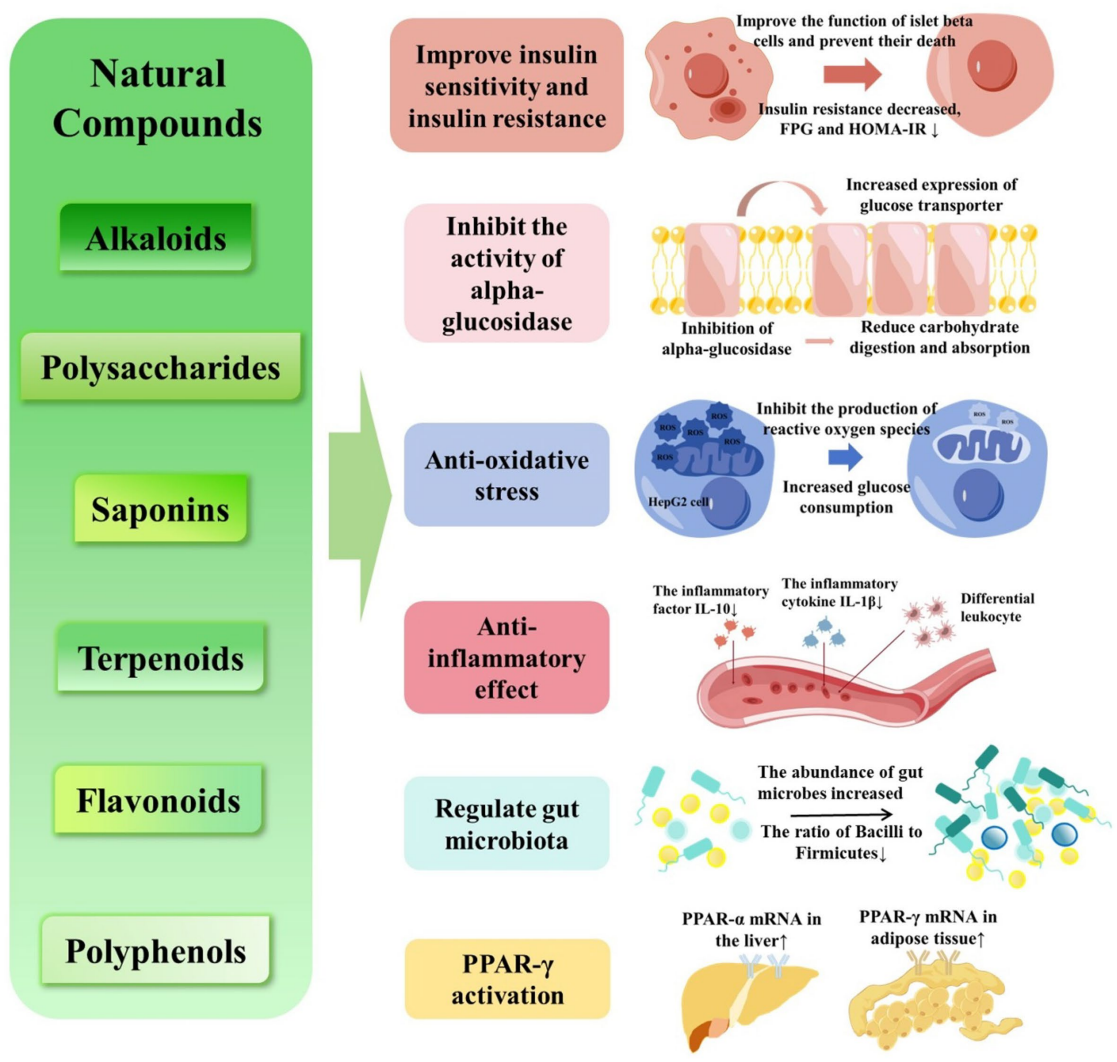
Mechanism of action for the effect of natural compounds.

Curcumin is a polyphenolic compound isolated from turmeric. Similar to the oral hypoglycemic drug thiazolidinedione, curcumin can activate PPAR-γ, thereby reducing blood sugar levels. PPAR-γ activation leads to suppression of gluconeogenesis and enhancement of glucose uptake and insulin sensitivity (44). Banaba is a deciduous tropical tree containing several polyphenolic compounds. Choi et al. (45) compared the hypoglycemic actions of banaba extract and soybean leaf extract in patients with prediabetes for 12 weeks. Both extracts similarly reduced HbA1c levels. In addition, patients treated with either extract displayed significantly reduced levels of baseline-adjusted final FPG and HOMA-IR compared with the control group. The hypoglycemic effect of banaba seems to reflect multiple mechanisms, including enhanced insulin sensitivity, by improving the expression of liver PPAR-α mRNA and adipose tissue PPAR-γ mRNA, thus reducing the risk of IGT progressing toward overt diabetes (Figure 1).

## 4 Conclusions and outlook

Our findings suggest a role for some natural compounds, such as Berberis, Curcumin, *Panax ginseng* and Oleanolic acid, in the management of prediabetes. With a large number of phenolic compounds, flavonoids, terpenoids, alkaloids, glycosides, quinones, lactones, and saponins, natural compounds have varying biological and pharmacological activities for prediabetes. The mechanism of prediabetes treatment by natural compounds might be related to decreased IR, reduced lipogenesis, oxidative stress, and inflammation, and improved function of β-cells function. While natural compounds have great potential for the treatment of prediabetes, current research and applications are relatively limited. Further studies focusing on the mechanism of action and active ingredients of natural compounds need to be performed using modern scientific and technological means to provide evidence-based support for their rational clinical application. In addition, a systematic safety review, including the safety and efficacy of natural compounds, requires further improvement. The present review provides the theoretical basis and fundamental scientific data for further research and the application of natural compounds in the treatment of prediabetes.

## Author contributions

JC: Writing – original draft. LJ: Writing – original draft. MC: Writing – original draft. KX: Writing – original draft. BH: Writing – original draft, Writing – review & editing. QH: Writing – review & editing.
